# Applications of Directed Self-Assembly Lithography in Advanced Semiconductor Devices

**DOI:** 10.3390/ma19214749

**Published:** 2026-11-09

**Authors:** Jiale Tang, Zhuohan Wang, Yuanlang Hou, Hanxiao Lu, Jiemin Zhang, Xiangshun Geng, Tian-Ling Ren

**Affiliations:** School of Integrated Circuits & Beijing National Research Center for Information Science and Technology (BNRist), Tsinghua University, Beijing 100084, China; tangjl26@mails.tsinghua.edu.cn (J.T.);

**Keywords:** block copolymer, directed self-assembly, advanced lithography, advanced semiconductor devices

## Abstract

As semiconductor devices continue to scale down, conventional lithography technologies face increasingly severe challenges in resolution, stochastic defect control, and manufacturing cost. Block copolymer (BCP) directed self-assembly (DSA), which offers molecular-scale control over pattern formation and compatibility with existing lithographic processes, has emerged as a promising complementary nanopatterning technology for advanced semiconductor manufacturing. This review summarizes the microphase-separation mechanisms of DSA, major guiding strategies including graphoepitaxy and chemoepitaxy, and pattern-transfer techniques such as dry and wet etching and sequential infiltration synthesis (SIS). In terms of device applications, particular emphasis is placed on recent progress in DSA for Fin Field-Effect Transistor (FinFET) fin patterning, silicon nanostructures, high-density contact holes, memory devices, two-dimensional semiconductors, and Complementary Metal–Oxide–Semiconductor (CMOS) optoelectronic devices. Key challenges, including defect control, process-window optimization, pattern transfer, and wafer-scale manufacturing, are further discussed, together with future directions involving the integration of DSA with extreme ultraviolet (EUV) lithography, advanced etching, and data-driven process optimization. This review aims to outline the technological evolution of DSA from nanoscale self-assembly toward integration into advanced semiconductor manufacturing and to provide a reference for high-resolution patterning at advanced technology nodes.

## 1. Introduction

Since the invention of the integrated circuit in 1958, the semiconductor industry has largely followed Moore’s law, achieving improved chip performance and reduced cost per function through continuous transistor scaling [1]. However, as device dimensions have entered the nanoscale regime, conventional optical patterning technologies have increasingly been constrained by both fundamental physical limits and manufacturing costs. When transistor critical dimensions are scaled below 10 nm, optical diffraction, increasing process complexity, and the cost burden associated with multiple patterning have become major challenges to further device scaling.

In recent years, advances in extreme ultraviolet (EUV) lithography have significantly accelerated manufacturing at advanced technology nodes. Nevertheless, EUV lithography still faces several challenges, including high equipment costs, difficulties in mask defect control, and stochastic effects. Owing to photon shot noise and the nonuniform distribution of components in chemically amplified resists, EUV-exposed patterns may suffer from critical dimension uniformity (CDU), line edge roughness (LER), and stochastic defects such as bridging and line breaks, thereby limiting further scaling toward more advanced nodes [2,3]. Meanwhile, although electron beam lithography (EBL) and nanoimprint lithography (NIL) offer high-resolution patterning capabilities, they remain constrained by factors such as throughput, template fabrication, defect control, and overlay accuracy, making it difficult for either technique alone to meet the requirements of large-scale integrated manufacturing.

Against this background, directed self-assembly (DSA) of block copolymers (BCPs) has attracted considerable attention as a patterning strategy that combines BCP microphase separation with prepattern-guided assembly to control nanoscale structures. DSA exploits the thermodynamic incompatibility between different blocks in BCPs to form ordered nanostructures with nanoscale periodicity through microphase separation, while physical or chemical guiding templates are used to control pattern placement [4]. Compared with conventional lithography, DSA offers several advantages, including material-defined resolution, pattern density multiplication, and potential defect-correction capabilities. It can therefore be used to generate regular periodic structures at sub-lithographic dimensions while reducing the need for some high-resolution exposure and multiple-patterning steps.

In recent years, the focus of DSA research has gradually shifted from early pattern density multiplication toward pattern enhancement and process integration. On the one hand, the development of high-χ block copolymer systems has extended DSA toward the sub-10 nm regime. On the other hand, hybrid patterning strategies that combine DSA with EUV lithography can use EUV to define the initial pattern placement, while subsequent BCP microphase separation improves pattern uniformity and mitigates the impact of stochastic defects. In addition, pattern-enhancement techniques such as sequential infiltration synthesis (SIS) can further increase the etch contrast of BCP templates, thereby enabling high-fidelity pattern transfer.

Although DSA has demonstrated significant potential for patterning at advanced technology nodes, its large-scale industrial implementation still faces several challenges, including limitations associated with microphase-separation kinetics, defect control across large-area wafers, process-window optimization, the synthesis and batch consistency of BCPs and guiding-layer materials, and compatibility with established silicon complementary metal–oxide–semiconductor (CMOS) fabrication processes [5]. Therefore, a thorough understanding of DSA mechanisms, guiding strategies, pattern-transfer processes, and device-level applications is essential for advancing the technology from laboratory research toward practical integration in advanced semiconductor manufacturing.

**Figure 1:**
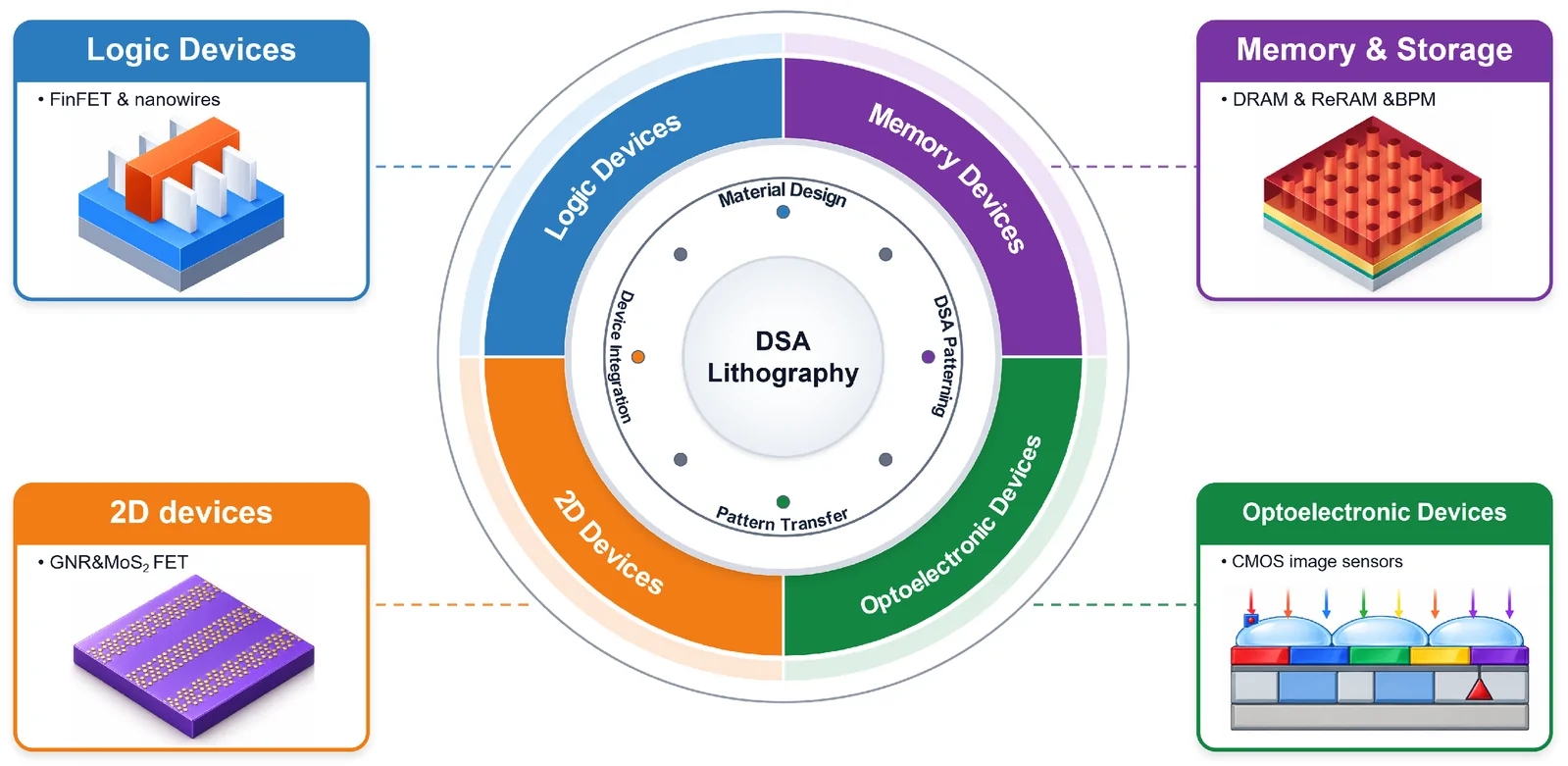
Overview of directed self-assembly (DSA) lithography, highlighting the interplay among material design, DSA patterning, pattern transfer, and device integration, together with representative applications in logic, memory and storage, two-dimensional devices, and optoelectronic devices.

This review systematically summarizes the development of DSA technology from fundamental mechanisms to device integration. First, the principles of block copolymer microphase separation and the key factors affecting self-assembled morphologies are introduced. Major patterning strategies, including graphoepitaxy, chemoepitaxy, and EUV-assisted DSA, are then discussed, followed by an overview of pattern-transfer techniques such as wet and dry etching and SIS. In terms of applications, particular emphasis is placed on DSA in advanced logic devices, including the patterning of fin field-effect transistor (FinFET) fins, silicon nanostructures, and high-density contact holes, together with recent progress in emerging areas such as memory devices, two-dimensional semiconductors, and CMOS optoelectronic devices. Finally, the key technical challenges currently facing DSA are discussed, and future directions involving its integration with EUV lithography, high-χ BCP materials, advanced etching technologies, and intelligent process control are highlighted. Figure 1 provides an overview of the key process modules and representative application areas of DSA lithography discussed in this review. Existing reviews, including Cheng et al. [5], provide broad accounts of DSA materials, processing, and applications. Building on these accounts, the present review emphasizes two linked questions for semiconductor integration: what geometry and pattern quality were achieved and whether functional devices were electrically tested. This device-integration perspective is supported by the quantitative comparison in Table 1 and the assessment of scalability and application relevance in Section 5.

## 2. Principles of DSA Lithography

### 2.1. Principles of Microphase Separation

**Figure 2:**
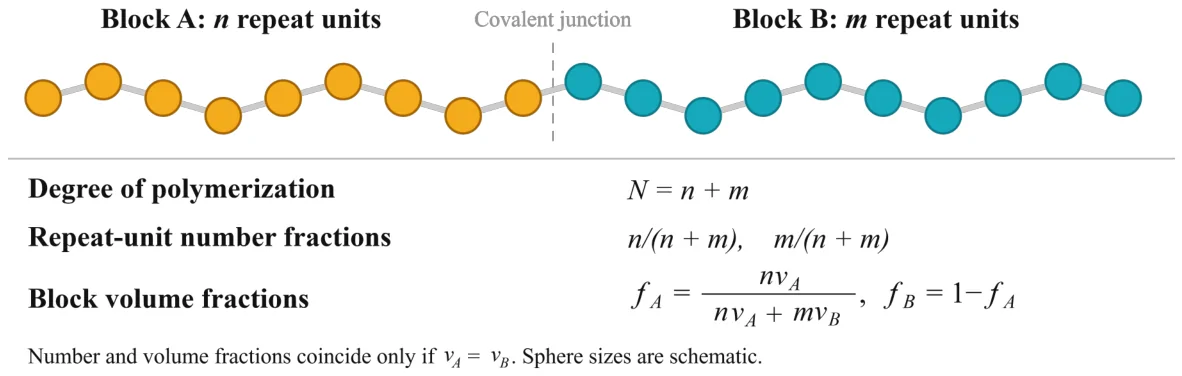
Schematic of an A-block-B diblock copolymer.

Block copolymers are a class of polymeric materials composed of two or more chemically distinct polymer segments covalently linked together. Among them, diblock copolymers (A-block-B) represent one of the simplest structures and are among the most widely used systems in DSA. As shown in Figure 2, a typical diblock copolymer consists of two chemically distinct homopolymer blocks connected by a covalent bond. The total degree of polymerization, N=n+m, and the block volume fractions, $f_{A}$ and $f_{B}$, are key parameters governing microphase separation and the self-assembled morphology. Here, *n* and *m* denote the numbers of repeat units in blocks A and B, and $v_{A}$ and $v_{B}$ denote their respective repeat-unit volumes. Assuming additive block volumes, $f_{A}=nv_{A}/\left(nv_{A}+mv_{B}\right)$ and $f_{B}=mv_{B}/\left(nv_{A}+mv_{B}\right)=1-f_{A}$. The repeat-unit number fractions, n/(n+m) and m/(n+m), coincide with these volume fractions only when $v_{A}=v_{B}$. Unlike conventional polymer blends, the different blocks in a BCP may exhibit strong thermodynamic incompatibility, but complete macrophase separation is prevented by their covalent linkage. Consequently, to minimize the free energy of the system, BCPs undergo microphase separation on the nanoscale, forming ordered nanostructures with characteristic periodicity.

The microphase separation behavior of BCPs is mainly governed by the Flory–Huggins interaction parameter (χ) between different blocks and the degree of polymerization (*N*), with the segregation strength generally characterized by the product χN. The parameter χ reflects the chemical incompatibility between different blocks, and a larger χ value indicates a greater difference in the interactions between the constituent segments. In contrast, N is associated with the polymer chain length. When χN is below the order–disorder transition threshold, the BCP system remains in a disordered state. As χN increases beyond the critical value, the polymer chains rearrange to form stable and periodic microphase-separated structures. The intrinsic periodicity, or pitch, of the BCP morphology is commonly denoted as $L_{0}$, and its value is closely related to the molecular weight, block composition, and interaction strength.

**Figure 3:**
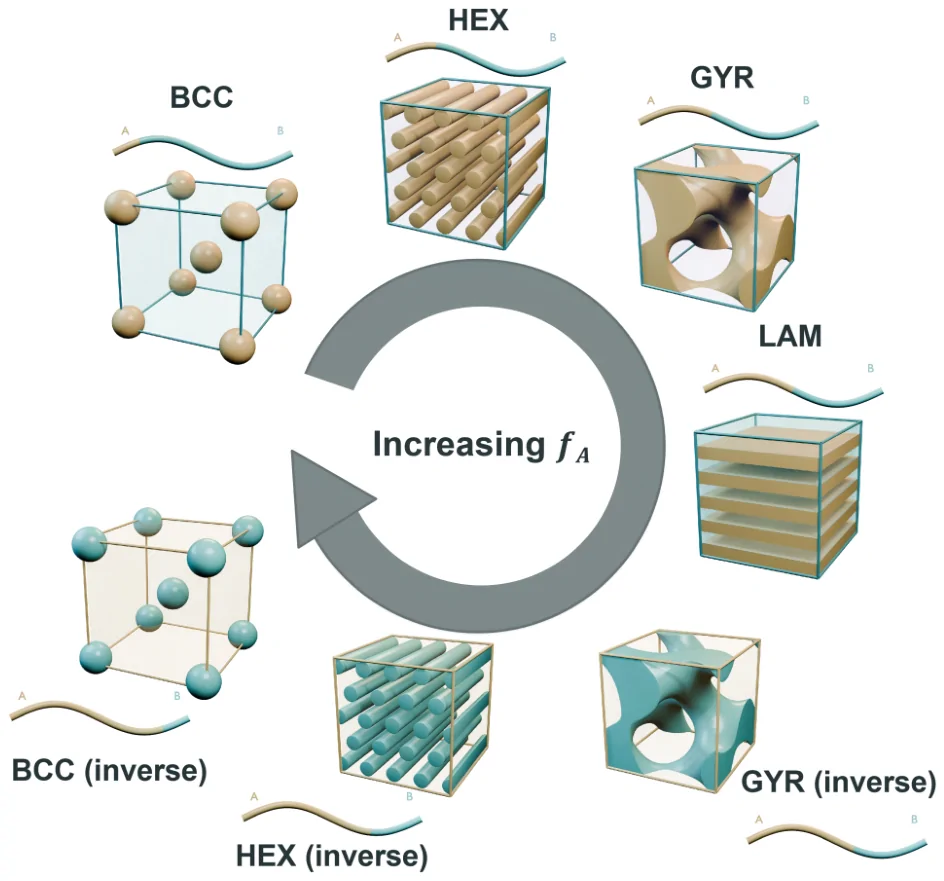
Representative bulk microphase-separated morphologies of A-block-B diblock copolymers with increasing volume fraction of block A ($f_{A}$), showing the transition from BCC spheres through HEX cylinders and GYR networks to LAM structures, followed by the corresponding inverse morphologies.

According to self-consistent field theory (SCFT), BCPs can form a variety of equilibrium morphologies depending strongly on the relative volume fraction of each block. As shown in Figure 3, variation in the volume fraction of block A ($f_{A}$) gives rise to a sequence of characteristic bulk microphase-separated structures, including the body-centered cubic spherical phase (BCC), hexagonally packed cylindrical phase (HEX), bicontinuous gyroid phase (GYR), and lamellar phase (LAM), followed by the corresponding inverse morphologies as block A becomes the majority component. Near symmetric compositions, the system generally favors a lamellar morphology, whereas increasingly asymmetric compositions favor gyroid, cylindrical, and eventually spherical microdomains. In practice, the equilibrium morphology and phase boundaries are governed not only by the block volume fraction but also by other factors, including the Flory–Huggins interaction parameter χ, molecular weight, polymer chain architecture, and interfacial interactions [12,13,14].

In DSA applications, cylindrical and lamellar morphologies are the most commonly employed structures. Vertically oriented cylindrical or lamellar microdomains can form continuous nanoscale channels or line/space (L/S) patterns extending through the film thickness, thereby facilitating the selective removal of one block and enabling high-contrast pattern transfer. Cylindrical morphologies are generally suitable for the fabrication of contact holes, dot arrays, and high-density hole patterns, whereas lamellar morphologies are more appropriate for the formation of L/S patterns. Therefore, controlling the BCP composition, molecular weight, and guiding template conditions to achieve the desired morphology and orientation is fundamental to the application of DSA in advanced semiconductor patterning.

The final microphase-separated morphology of a BCP is determined not only by its intrinsic molecular parameters but also by processing conditions, including the composition of the neutral layer, anchoring agents, annealing temperature, and film thickness [15]. Conventional low-χ systems, such as polystyrene-block-poly(methyl methacrylate) (PS-*b*-PMMA), benefit from well-established synthesis and processing methods; however, their relatively weak intersegment interactions limit the achievable intrinsic periodicity, making them increasingly inadequate for sub-10 nm patterning at advanced technology nodes. Consequently, recent research has progressively shifted toward high-χ BCP systems. High-χ materials can maintain microphase separation at a lower *N*, enabling smaller intrinsic periods while retaining thermodynamically stable segregated domains [6]. However, increasing χN at a given chain length can increase the barriers to chain rearrangement and slow defect annihilation. Thus, high-χ material design and annealing conditions must balance pattern resolution, segregation strength, and assembly kinetics, as discussed further in Section 4.1.

### 2.2. Core Patterning Strategies for DSA

**Figure 4:**
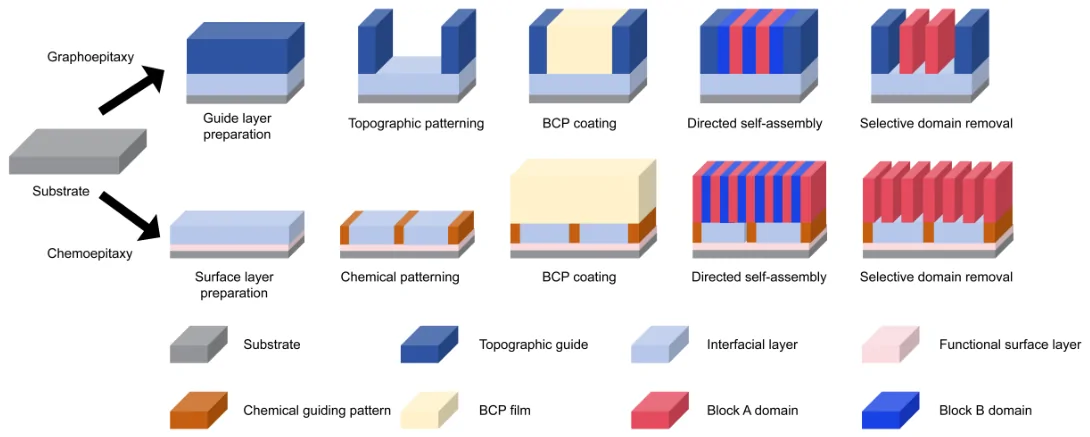
Schematic illustration of typical graphoepitaxy and chemoepitaxy processes in DSA.

Graphoepitaxy directs BCP assembly using topographic trenches or holes and their geometric confinement, whereas chemoepitaxy uses essentially planar patterns of chemical affinity toward the constituent blocks. Both involve interfacial wetting; their main distinction is topographic versus chemical guidance.As illustrated in Figure 4, the typical process flows of the two approaches are shown from guiding structure formation and BCP coating to self-assembly and subsequent pattern transfer.

#### 2.2.1. Graphoepitaxy

Graphoepitaxy is a DSA strategy in which pre-fabricated three-dimensional topographic templates are used to guide the self-assembly of BCPs. In this approach, lithographic techniques are typically employed to form guiding structures with defined geometries on the substrate surface, such as trenches or holes. The spatial confinement provided by these templates subsequently induces the directional alignment of BCP microdomains, thereby producing highly ordered nanopatterns [16,17,18]. The sidewalls and bottom surfaces of the templates may exhibit preferential interactions with different blocks, enabling control over the orientation of the BCP structures throughout the confined region. To minimize assembly defects, the template dimensions generally need to be commensurate with the intrinsic BCP period, $L_{0}$, and are often designed as integer multiples of $L_{0}$ [19].

The geometric confinement provided by graphoepitaxy offers a straightforward means of converting relatively low-density prepatterns into high-density nanostructures. For example, physical hole templates can be defined using 193 nm immersion lithography (193i), followed by graphoepitaxial self-assembly of cylindrical BCPs to achieve hole-pattern multiplication. This approach can extend the patterning capability of 193i toward advanced technology nodes while reducing the need for some multiple-patterning and mask steps [20].

The use of physical templates also has certain limitations. These templates occupy part of the wafer surface, thereby reducing the effective area available for patterning, while the geometric confinement imposed by template boundaries may induce localized stress and lead to distortion, displacement, or defect formation in BCP microdomains near the edges. In addition, the quality of the final patterns remains affected by factors such as template roughness, dimensional variations, and BCP–template interfacial interactions. Therefore, appropriate template dimension design, surface-property control, and matching between the template geometry and the intrinsic BCP periodicity are critical for achieving high-quality graphoepitaxial DSA.

In recent years, graphoepitaxy has demonstrated renewed application potential in specific advanced patterning scenarios through the development of various process-control and optimization strategies. For contact-hole shrink applications, Wu et al. employed cylindrical PS-*b*-PMMA BCPs in combination with physical hole templates and optimized the microphase separation behavior by adjusting the BCP/homopolymer ratio, achieving stable hole-size reduction and the formation of ordered hole arrays [21]. With the development of high-χ BCPs, graphoepitaxy has been further extended toward smaller dimensions and higher assembly efficiency. Putranto et al. used a silicon-containing high-χ block copolymer, poly(1,1-dimethylsilacyclobutane)-block-polystyrene (PDMSB-*b*-PS), to achieve vertically oriented lamellar structures with a 9 nm half-pitch in plasma-modified spin-on-carbon trench templates, while interfacial wetting control was employed to improve orientation stability under three-dimensional confinement [22]. Hu et al. accelerated the self-assembly of PS-*b*-PMMA within physical hole templates using rapid solar thermal annealing, producing contact-hole structures with dimensions of approximately 20 nm and demonstrating the potential of rapid annealing for improving assembly efficiency [23].

Overall, graphoepitaxy remains highly valuable for contact-hole shrinkage, high-density periodic arrays, and the fabrication of specialized nanostructures because of its well-defined geometric confinement. However, for large-area fabrication of advanced logic devices, further scaling is constrained by the surface area occupied by physical templates and their limited compatibility with complex three-dimensional structures. In contrast, chemoepitaxy offers better compatibility with planar lithographic processes and enables wafer-scale pattern guiding. Consequently, it has increasingly emerged as an important approach for EUV–DSA hybrid patterning strategies in recent years.

#### 2.2.2. Chemoepitaxy

Unlike graphoepitaxy, which relies on three-dimensional geometric confinement, chemoepitaxy controls BCP orientation by selectively modifying the substrate surface to create guiding regions with different surface energies or preferential affinities toward specific blocks. The selective interfacial interactions between the individual BCP blocks and the substrate are thereby utilized to direct the orientation of the microdomains. Because the chemical guiding patterns remain essentially planar, chemoepitaxy offers good compatibility with planar processing and is more suitable for constructing high-density guiding patterns over large wafer areas. As a result, it has gradually become an important direction in the development of advanced DSA processes.

The assembly quality of chemoepitaxy is highly dependent on the interfacial matching between the guiding patterns and the BCP material. In addition to the pitch and width of the guiding stripes, differences in surface energy, brush-layer composition, and the penetration behavior of polymer segments into the interfacial layer can all affect the final orientation stability and defect density. Only when the interfacial interactions between the guiding layer and the BCP are maintained within an appropriate range can sufficient pattern confinement be balanced with adequate chain-segment mobility, thereby enabling the formation of stable, well-ordered structures with a low defect density [24].

In recent years, high-χ BCP systems have further extended the resolution limit of chemoepitaxy. Maekawa et al. employed a high-χ PS-*b*-PMMA-derived block copolymer and, by optimizing the dimensions of the chemical guiding patterns and the thermal annealing conditions, achieved highly reliable vertically oriented lamellar structures and sub-10 nm half-pitch line patterns on 300 mm silicon wafers. The occurrence of parallel-oriented regions and dislocation defects was also significantly reduced [6], providing a process foundation for advanced lithographic technologies.

#### 2.2.3. Hybrid EUV–DSA Patterning Strategies

As semiconductor manufacturing continues to advance toward more aggressive technology nodes, EUV lithography has become one of the key technologies for high-resolution pattern definition. However, with continued dimensional scaling, EUV lithography faces increasing challenges associated with photon shot noise, material fluctuations, and the limitations imposed by ultrathin photoresists, leading to degraded LER and local critical dimension uniformity (LCDU), increased stochastic defects, and reduced etch margins. Consequently, hybrid patterning strategies that combine the high-precision pattern-definition capability of EUV lithography with the pattern-rectification capability of DSA have gradually emerged as a representative approach for advanced patterning.

Verstraete et al. investigated the pattern-rectification capability of DSA for EUV-defined L/S and hexagonal contact-hole (HEXCH) patterns. In this approach, EUV lithography was first used to define the initial patterns, after which the pattern information was transferred to a chemical guiding layer through surface chemical treatment, followed by BCP self-assembly to reconstruct and rectify the patterns. For the HEXCH arrays, DSA rectification reduced the LCDU from 2.26 nm to 1.52 nm. Further optimization of the underlayer treatment and polymer film thickness also improved the pattern placement error (PPE) [3]. Van Bel et al. further investigated roughness and defect behavior during EUV–DSA processing of 28 nm-pitch L/S patterns using EUV-defined chemical guiding patterns for 1× DSA. Their results showed that part of the roughness and perturbations present in the EUV prepatterns could be attenuated during self-assembly, resulting in improved control of LER and line-width roughness (LWR), together with faster assembly and defect-annihilation kinetics [25]. These results indicate that DSA is no longer limited to density multiplication, but can also serve as an effective approach for improving pattern quality and mitigating stochastic defects.

In addition, the development of high-χ BCP materials has provided an important materials platform for extending EUV–DSA toward smaller dimensions. Previous studies have shown that, by increasing the segregation strength and optimizing the chemical guiding conditions, good structural ordering and pattern stability can be maintained at sub-10 nm length scales [6].

Overall, EUV lithography and DSA exhibit strong complementarity: EUV provides high-resolution initial patterns, while DSA improves pattern roughness, dimensional variation, and certain defect types through BCP self-assembly. In addition, some silicon-containing BCPs can provide enhanced etch resistance [13,26], further extending the role of EUV–DSA from pattern rectification to improved pattern-transfer performance.

### 2.3. Pattern Transfer

To replicate the periodic patterns generated by microphase separation into a hard mask or a target functional material layer, subsequent pattern-transfer steps, including selective block removal, mask enhancement, and anisotropic etching, are required. The pattern-transfer capability is therefore a critical factor in determining whether DSA can be successfully implemented in semiconductor device fabrication.

Conventional BCP pattern-processing methods mainly include wet etching [27,28], ultraviolet/ozone (UV/$O_{3}$) selective removal [27,28], plasma etching [29], and inductively coupled plasma (ICP) etching [30,31,32]. Wet etching offers the advantages of process simplicity and relatively low cost and can be used for the selective removal of specific polymer blocks. However, for fine nanoscale structures with high aspect ratios, it may lead to lateral dimensional changes or structural damage. UV/$O_{3}$ treatment is also commonly employed to degrade or remove selected BCP microdomains, thereby generating polymer templates with sufficient etch contrast. Nevertheless, its primary function remains selective block removal, and it is generally insufficient on its own to achieve high-fidelity pattern transfer.

Block copolymer templates generally exhibit relatively low mechanical strength and limited resistance to plasma etching. To address these limitations, SIS has recently been introduced into DSA processes as an effective template-enhancement strategy. SIS utilizes the selective infiltration and reaction of vapor-phase precursors within specific BCP domains to generate inorganic components, such as ${\mathrm{Al}}_{2}O_{3}$, in situ within the polymer microdomains. In this manner, the original polymer patterns can be converted into inorganic masks with improved mechanical stability and higher etch selectivity. This approach can significantly enhance the stability of ultrathin BCP patterns during subsequent plasma processing while reducing the risks of pattern collapse, line breakage, and morphological distortion. Recent studies have demonstrated that the combination of DSA and SIS enables pattern transfer of high-density sub-10 nm features and high-aspect-ratio silicon nanostructures [9]. After a polymer or inorganic hard mask with sufficient etch resistance has been formed, dry-etching techniques, such as reactive ion etching (RIE) or ICP etching, can be employed to further transfer the patterns into target material layers, including Si, ${\mathrm{SiO}}_{2}$, and SiN [30,31,32]. Therefore, the combination of SIS-based inorganic template enhancement and highly anisotropic ICP-RIE provides a more reliable process route for the high-fidelity transfer of BCP nanopatterns into target material layers [9].

### 2.4. AI Use of Images

The Figure 1 and Figure 3 were generated using [OpenAI Codex] on [gpt-5.6-sol]. Figure 1 was based on the authors’ earlier schematics. No image elements were copied from external websites and stitched into these device illustrations. The tool was used to assist with drawing and layout, and its image-generation tool was used to generate and edit the device illustrations. For Figure 3, AI with the recorded assistant model identifier “gpt-5.6-sol” assisted in writing Python scripts. The morphological schematics were then modeled and rendered using Blender 5.0.0. This was an AI-assisted programming and procedural rendering workflow, rather than direct text-to-image generation of the morphological models. Reference schematics from publications or websites were provided to communicate the intended block-copolymer morphology types. The final three-dimensional models were constructed programmatically in Python and rendered in Blender. The reference images themselves were not pasted into the rendered morphology panels. The generated outputs were subsequently edited and arranged using WPS Office for annotation, scaling, and formatting. All AI-generated content was manually reviewed by the authors to ensure accuracy and consistency with the cited literature.

## 3. Applications of DSA in Advanced Semiconductor Devices

### 3.1. Applications of DSA in Logic Devices

For the patterning of critical fin and nanowire arrays in advanced logic devices, DSA has demonstrated clear potential for device integration. As early as 2005, Black et al. reported one of the earliest device-level demonstrations of DSA in multi-nanowire Si FETs. The fabricated silicon nanostructures had cross-sectional dimensions of approximately 15 nm × 15 nm with an array pitch of 40 nm [33]. This work demonstrated that BCP self-assembled patterns can be used not only for the fabrication of nanoscale structures but also for integration with transistor manufacturing processes, thereby laying the foundation for subsequent applications of DSA in advanced logic devices.

With the widespread adoption of FinFETs, converting the one-dimensional periodic L/S patterns generated by DSA into the two-dimensional structures required for practical circuit layouts has become a key challenge for further implementation. In 2014, Tsai et al. employed a PS-*b*-PMMA system together with a graphoepitaxial strategy, in which two-dimensional geometrical features were introduced into physical guiding templates. By exploiting the preferential interfacial affinity between hydrogen silsesquioxane (HSQ) sidewalls and the PMMA block, controlled local morphological transitions were induced at predefined locations, enabling line-end truncation and the formation of specific pattern breaks. Combined with an organic planarization layer-assisted tone-inversion pattern-transfer process, this approach enabled the fabrication of silicon fin arrays with a 29 nm pitch on silicon-on-insulator substrates, followed by the successful fabrication of functional FinFET devices [34]. Under a gate-first high-k metal gate integration scheme, the fabricated multi-fin transistors exhibited good short-channel control, with a subthreshold swing of approximately 70 mV/dec and an on/off current ratio of approximately $10^{5}$. This work demonstrated that DSA is capable not only of generating regular one-dimensional periodic structures but also of providing a certain degree of two-dimensional pattern control through template design, thereby extending its applicability to practical logic-device layouts. Subsequently, the same research group employed a high-χ BCP system to further reduce the fin pitch to 24 nm, demonstrating the potential of DSA for continued FinFET fin scaling [35].

**Figure 5:**
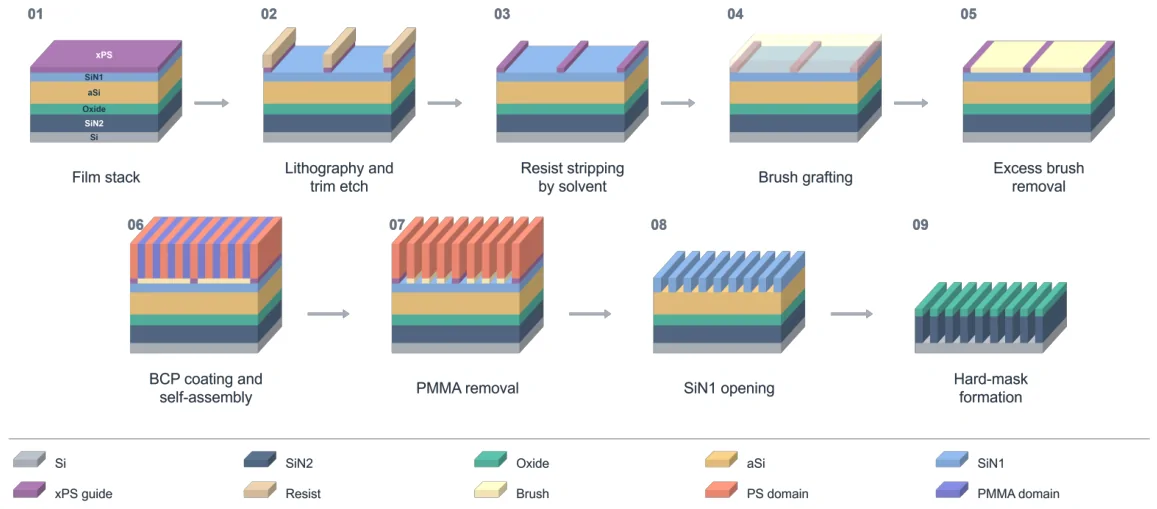
Main process flow for the fabrication of FinFET fins using chemoepitaxial DSA. (01) Initial film stack comprising cross-linked polystyrene (xPS), an upper silicon nitride layer (SiN1), amorphous silicon (aSi), oxide, a lower silicon nitride layer (SiN2), and a Si substrate. (02–03) Lithographic definition and trim etching of the xPS guides, followed by solvent stripping of the resist. (04–05) Brush grafting and removal of excess brush to establish the background surface. (06) PS-*b*-PMMA coating and directed self-assembly. (07) Selective PMMA removal. (08–09) Opening of SiN1 and transfer through the underlying stack to form the hard mask for subsequent fin patterning.

In studies conducted under conditions more representative of advanced-node manufacturing, Liu et al. employed chemoepitaxial DSA on a 300 mm wafer development platform to fabricate FinFET fin arrays targeting the 7 nm technology node and systematically compared the process with a self-aligned quadruple patterning (SAQP) baseline, with the main process flow shown in Figure 5. Morphological characterization showed similar CDU, whereas the mean LER/LWR values were 1.38/1.26 nm for DSA and 0.78/0.98 nm for the SAQP baseline. For 24-fin long-channel n-channel FinFETs with a gate length of approximately 500 nm, the median linear threshold voltages were approximately 0.176 V (DSA) and 0.166 V (SAQP), and the median linear subthreshold swings were 62.1 and 62.9 mV/dec, respectively. For short-channel devices with an approximately 15 nm gate length, the study reported no statistically significant differences in threshold voltage and subthreshold swing, while a small but statistically significant difference in drain-induced barrier lowering (DIBL) was observed and attributed by the authors mainly to wafer-to-wafer process variations [7]. These results support comparable performance for the tested devices and metrics.

Figure 5 outlines the corresponding patterning sequence. An xPS guiding layer is first deposited on the SiN1/aSi/oxide/SiN2/Si stack. Lithography and trim etching define the xPS stripes, after which the resist is stripped. A brush is grafted onto the exposed background, and excess brush is removed to establish the chemical contrast required for assembly. PS-*b*-PMMA is then coated and annealed to form aligned lamellae. Selective PMMA removal leaves a PS line mask, which is used to open SiN1 and transfer the dense line pattern through the underlying stack.

In addition to FinFET fins, DSA has also been explored for the fabrication of sub-10 nm nanowire templates. Qiu et al. combined EBL-defined HSQ guiding structures with polystyrene-block-poly(dimethylsiloxane) (PS-*b*-PDMS) DSA to fabricate highly oriented nanowire arrays with linewidths of approximately 9–11 nm [36]. These results demonstrate that the synergistic control provided by high-χ BCPs and prepatterned guiding structures can further extend DSA toward sub-10 nm one-dimensional silicon nanostructures, providing a feasible fabrication strategy for the patterning of silicon nanowire (SiNW) transistors and other nanowire-based devices.

To improve the mechanical stability and etch resistance of BCP templates, Chen et al. combined DSA with SIS, selectively incorporating ${\mathrm{Al}}_{2}O_{3}$ into the polymer microdomains to form an inorganic hard mask. Subsequent pattern transfer by etching yielded silicon nanostructures with a top linewidth of approximately 6.6 nm, a pitch of 28 nm, and a height of approximately 53.5 nm. The subsequently fabricated junctionless FinFETs (JL-FinFETs) exhibited good gate electrostatic control, with a subthreshold swing of 69.59 mV/dec and an on/off current ratio exceeding $10^{7}$ [9]. This study demonstrates that the integration of DSA with SIS can enhance the pattern-transfer capability of sub-10 nm features and support device-level fabrication of high-aspect-ratio silicon nanostructures.

The application of DSA in advanced logic devices has expanded to include two-dimensional pattern control of FinFET fins, process integration for the 7 nm technology node, fabrication of sub-10 nm SiNWs, and high-density contact-hole/via patterning. Existing studies indicate that the advantages of DSA extend beyond dimensional scaling, particularly in enabling low-defect and high-fidelity pattern transfer of dense, regular features. For further implementation in high-volume logic manufacturing, however, several challenges must still be addressed, including defect density, pattern placement and overlay accuracy, and compatibility with CMOS process flows.

### 3.2. Applications of DSA in Memory Devices

#### 3.2.1. DRAM and High-Density Contact/via Patterning

**Figure 6:**
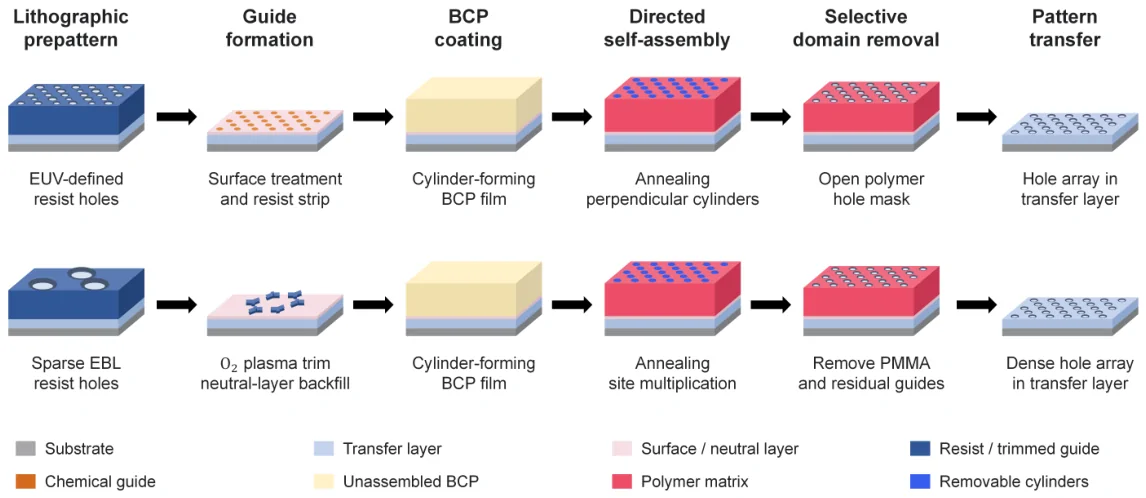
Comparison of two DSA process routes for dense contact-hole patterning.

The continued scaling of dynamic random-access memory (DRAM) relies heavily on the precise patterning of high-density contact holes, capacitor arrays, and related periodic structures. As DRAM nodes continue to shrink, conventional lithography combined with multiple-patterning techniques faces increasingly complex process flows, rising fabrication costs, and the accumulation of patterning errors, including LER, pitch walking, and local dimensional variations. DSA has therefore been regarded as a promising complementary patterning technology for advanced DRAM scaling. Figure 6 schematically contrasts EUV-defined chemical guidance for contact-hole pattern rectification with a plasma-trimmed template route for density multiplication.

In the top-row process of Figure 6, EUV lithography first defines resist holes at the intended locations. Surface treatment followed by resist stripping converts the lithographic information into chemical guiding sites on the transfer stack. A cylinder-forming BCP is coated and annealed so that perpendicular cylindrical domains assemble in registry with these guides. Selective removal of the cylindrical domains opens holes in the remaining polymer matrix, which then serves as a mask for transfer into the underlying layer. The role of DSA in this route is to improve the local size uniformity and placement of the EUV-defined hole pattern while retaining lithographically specified positions; the general integration approach is illustrated by the EUV–DSA studies discussed here [8,37,38].

The bottom-row process of Figure 6 instead begins with a sparse EBL-defined hole pattern. $O_{2}$ plasma trimming reshapes the resist into guiding features, while neutral-layer backfilling establishes the surrounding surface conditions for assembly. After BCP coating and annealing, additional cylindrical domains form between the sparse guides. Removing the PMMA domains and residual guides opens a dense hole mask for subsequent pattern transfer [10]. In the study by Zhou et al., the reported 48× overall areal density multiplication is defined relative to the initial lithographic hole array: template trimming contributes 2×, while subsequent assembly contributes 24× relative to the trimmed guiding-feature density.

Early studies primarily focused on the capability of DSA to achieve pattern density multiplication in DRAM. Liu et al. developed a chemical prepatterning method for the directed assembly of PS-*b*-PMMA, laying the foundation for subsequent chemoepitaxial density-multiplication processes [39]. Subsequently, Bao et al. proposed a pattern multiplication scheme targeting 15 nm NAND and 2× nm DRAM [40]. Kamon et al. further evaluated the potential integration of DSA into 14 nm DRAM using virtual fabrication methods. Their results indicated that DSA could potentially eliminate certain steps in SAQP and, to some extent, expand the patterning process window [41]. Collectively, these studies demonstrate that the early value of DSA in DRAM was primarily associated with increasing pattern density while reducing reliance on complex multiple-patterning schemes.

Further scaling of DRAM will increasingly depend on the synergistic integration of high-χ BCPs, novel chemical guiding templates, EUV exposure, and high-fidelity pattern-transfer technologies to simultaneously meet the stringent requirements for local dimensional uniformity, placement accuracy, defect density, and manufacturing cost.

#### 3.2.2. Emerging Nonvolatile Memories

Beyond conventional high-density memories such as DRAM, DSA-assisted patterning has also been applied to dimensional scaling and structural engineering in emerging nonvolatile memories. Unlike approaches in which BCPs directly serve as functional materials, these studies typically exploit periodic nanostructures formed through self-assembly to define electrodes, resistive-switching cells, or phase-change contact regions, thereby integrating DSA into memory fabrication processes as a high-resolution patterning technique.

In resistive random-access memory (ReRAM), nanopatterning can be used to construct high-density resistive-switching cells and spatially confine the regions in which conductive filaments form. Frascaroli et al. fabricated nanoelectrode arrays using BCP templates, enabling high-density ${\mathrm{HfO}}_{2}$-based ReRAM structures [42]. Park and You et al. employed ${\mathrm{SiO}}_{x}$ nanostructures derived from silicon-containing BCPs to spatially confine the resistive-switching regions and conductive pathways, thereby improving device uniformity and local switching behavior [43,44]. These results indicate that DSA primarily controls the geometry of nanoelectrodes and active regions and can thereby influence conductive-filament formation and resistive switching. The stored state in these ReRAM devices is encoded by a resistance change associated with the formation, rupture, or modification of conductive pathways.

In phase-change memory (PCM), DSA can likewise be used to construct ultrasmall contact structures. Tung et al. employed PS-*b*-PMMA DSA to form contact patterns with dimensions of approximately 20 nm and integrated them into the PCM fabrication process as localized contact regions between the metallic bottom electrode and the Ge_2_Sb_2_Te_5_ phase-change material [45]. Because the programming current of phase-change memory is closely related to the effective phase-change volume and contact dimensions, reducing the contact area through DSA provides a potential route toward decreasing cell dimensions and power consumption.

#### 3.2.3. Bit-Patterned Media

Beyond semiconductor memories, bit-patterned media (BPM) represent another class of high-density information-storage applications in which DSA attracted considerable attention at an early stage. BPM discretizes a continuous magnetic thin film into individual nanoscale magnetic islands to increase magnetic recording density and mitigate the stability limitations associated with superparamagnetic effects. However, its fabrication requires simultaneous control over nanoscale feature dimensions, large-area periodic ordering, and high manufacturing throughput, making it difficult for EBL alone to achieve both high resolution and sufficient productivity. Owing to its capabilities for forming high-density arrays and rectifying pattern placement, BCP DSA represents a promising approach for BPM template fabrication.

Yang et al. employed the directed self-assembly of cylindrical PS-*b*-PMMA on chemically prepatterned substrates to form hexagonal nanopore arrays with a pitch of approximately 28 nm and demonstrated the potential advantages of DSA over direct EBL patterning in terms of dimensional and positional uniformity [46]. Griffiths et al. systematically discussed both graphoepitaxial and chemoepitaxial strategies for BPM fabrication and pointed out that, to balance patterning precision and manufacturing throughput, DSA should be integrated with high-throughput prepatterning techniques such as NIL [47].

In recent years, related research has further advanced toward the integration of pattern formation with functional-material fabrication. Zhang et al. proposed NIL-assisted metallized-BCP self-assembly strategies, in which NIL defines large-scale positional and layout information, BCP self-assembly establishes nanoscale ordering, and subsequent in situ pyrolysis forms $L1_{0}$-FePt magnetic bit islands [48,49]. This approach integrates prepatterning, BCP self-assembly, and magnetic-material fabrication, reflecting the evolution of DSA in high-density magnetic recording toward functional-material integration.

Taken together, DSA applications in DRAM have expanded from density multiplication to EUV pattern rectification, while in ReRAM and PCM, DSA has been employed to define nanoscale functional regions. In BPM, DSA has further been extended to the fabrication of high-density magnetic units. Its large-scale implementation, however, still requires further advances in defect control, placement accuracy, material compatibility, and large-area uniformity.

### 3.3. Applications of DSA in Emerging Semiconductor Devices

Beyond conventional silicon-based logic and memory devices, DSA has gradually been extended to two-dimensional materials, three-dimensional nanostructures, and optoelectronic functional devices. Although most of these studies remain at the proof-of-concept or device-prototype stage, they further demonstrate the potential of DSA as a versatile nanoscale patterning platform.

For two-dimensional electronic materials, DSA can be used to construct sub-10 nm nanopatterns and further enable dimensional scaling of transistor channels. Jeong et al. employed cylindrical PS-*b*-PDMS directed by the confinement of topographically patterned Au guides and transferred the resulting self-assembled patterns into graphene, producing graphene nanoribbons (GNRs) with widths of approximately 6 nm and multichannel GNR field-effect transistors. The process was further demonstrated on 6-inch Si/${\mathrm{SiO}}_{2}$ wafers, indicating the potential of BCP DSA for sub-10 nm patterning of two-dimensional materials and wafer-scale device integration [50].

**Figure 7:**
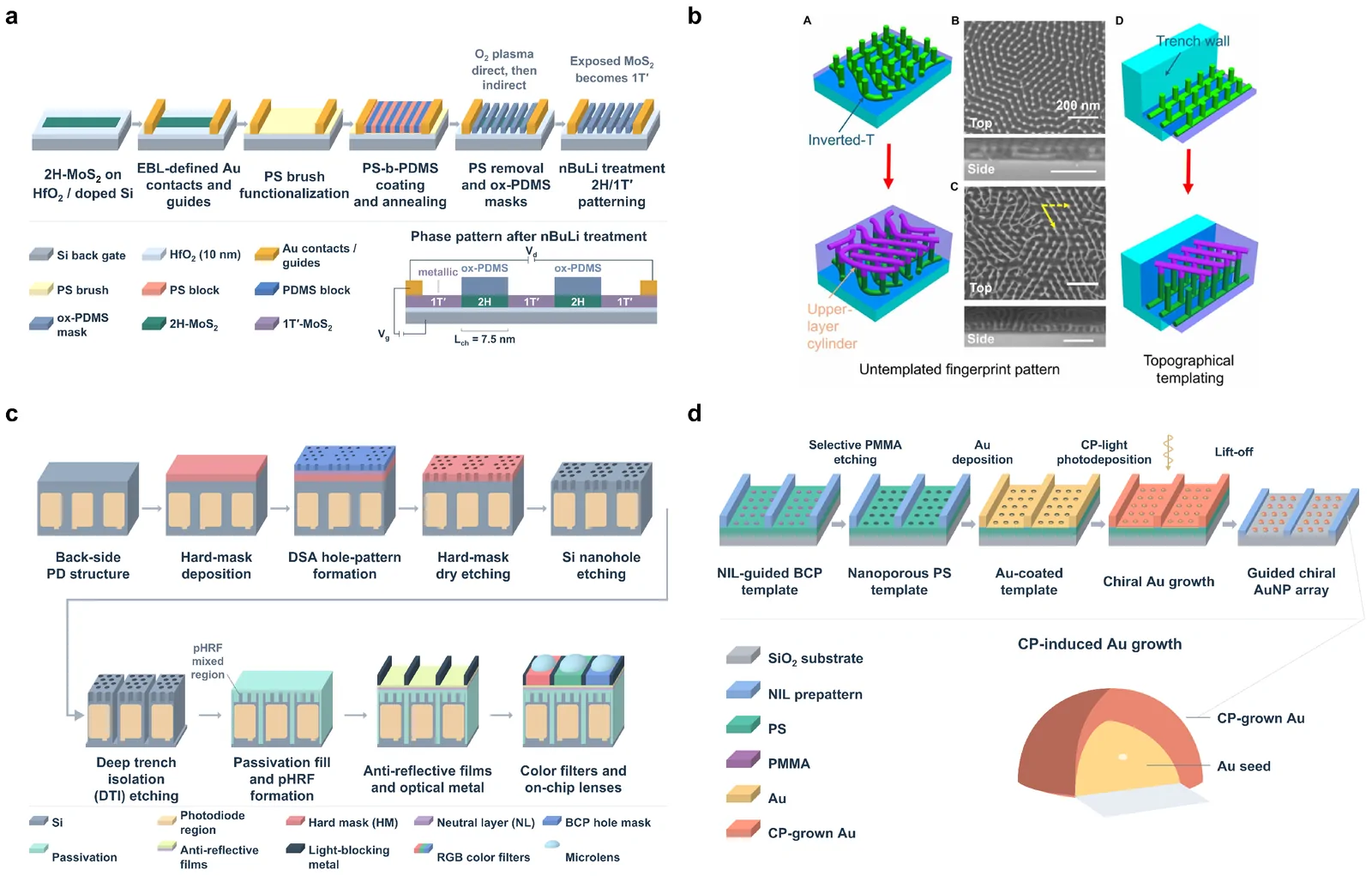
(**a**) Schematic illustration of DSA-enabled selective 2H-to-1T′ phase conversion in MoS_2_ and the formation of a series-connected chain of sub-10 nm-channel FETs. The inset shows a reduced number of repeating units for clarity. The reported device comprised six FETs in series. (**b**) Hierarchical construction of three-dimensionally interconnected BCP networks: (**A**) schematics showing the single-layer inverted-T structure (**top**) and the double-layer interconnected network (**bottom**) on a flat substrate; (**B**,**C**) top- and side-view scanning electron microscopy (SEM) images of the single- and double-layer structures, respectively; (**D**) schematics showing the corresponding ordered structures under topographic trench guidance [52]. (**c**) fabrication process of a back-illuminated CMOS image sensor using a DSA-fabricated pseudo-high-refractive-index film (pHRF); (**d**) schematic illustration of the synergistic NIL–DSA process for fabricating guided chiral Au nanoparticle arrays.

Beyond geometric dimension definition, DSA can also provide nanoscale masks for selective chemical treatment of two-dimensional semiconductors. In the process illustrated in Figure 7a, 2H-MoS_2_ is supported on an HfO_2_/doped-Si back-gate stack. EBL-defined Au contacts also provide topographic guidance, followed by PS-brush functionalization and PS-*b*-PDMS coating and annealing. Oxygen-plasma processing removes PS and converts the PDMS domains into oxidized-PDMS (ox-PDMS) masks. Subsequent *n*-butyllithium (nBuLi) treatment converts the exposed MoS_2_ into metallic 1T′ regions, while the protected 2H regions remain semiconducting channels. Nourbakhsh et al. used this selective phase-patterning approach to fabricate periodically alternating 2H/1T′ structures with channel lengths of approximately 7.5 nm; the adjacent metallic regions act as local source/drain contacts and access regions [51]. The process therefore uses DSA to control both the channel dimensions and the spatial distribution of electronic phases.

As device architectures continue to evolve from two-dimensional planar structures toward three-dimensional integration, DSA research has also expanded to complex nanonetworks with both in-plane and out-of-plane connectivity. Ma et al. proposed a hierarchical DSA strategy that combines topographic templates, BCP periodicity matching, and interdomain interactions between different self-assembled layers to construct three-dimensionally interconnected BCP structures, including ladder, orthogonal cross-point, and oblique cross-point architectures, as shown in Figure 7b. Figure 7b contrasts the interconnected networks formed without topographic templates with those aligned by trench confinement. In this approach, the self-assembled domains in the first layer further participate in positioning the structures in the second layer, thereby enabling self-aligned assembly between different layers [52]. It should be emphasized that this work primarily demonstrates the structural fabrication capability of three-dimensional interconnected BCP nanonetworks and has not yet realized fully functional cross-point memory or back-end interconnect devices.

In optoelectronic devices, DSA has also been demonstrated in integration schemes that more closely resemble practical device fabrication. Oshiyama et al. incorporated DSA into the fabrication process of a back-illuminated CMOS image sensor, using BCP self-assembly to form high-density nanopore arrays, followed by hard-mask processing and Si dry etching to construct a pseudo high refractive index film (pHRF), as illustrated in Figure 7c. As outlined in Figure 7c, a hard mask and a DSA hole mask are formed on the back-side photodiode structure. The hole pattern is transferred through the hard mask and into Si, followed by deep-trench-isolation etching. Passivation filling forms the mixed region associated with the pHRF, after which antireflective films, optical metal, color filters, and on-chip lenses complete the optical stack.The resulting nanopores had diameters of approximately 15 nm and a pitch of approximately 40 nm. Prototype-device measurements showed that, compared with the conventional structure, integration of the pHRF increased the quantum efficiency by approximately 9% in the blue-light region and approximately 4% in the green-light region. After optimization of the etching conditions, the dark current remained comparable to that of the baseline device [11]. This work therefore provides a representative device-level example demonstrating that periodic nanostructures generated by DSA can also be integrated into CMOS fabrication processes as functional optical interfaces.

The integration of DSA with other nanopatterning technologies has further expanded its applications in functional optical devices. Jung et al. combined NIL with PS-*b*-PMMA DSA, using NIL to define large-scale orientation and positional information while employing BCP self-assembly to provide periodic nanoscale templates, which were subsequently used to construct Au nanoparticle arrays, as shown in Figure 7d. Figure 7d summarizes the sequence: NIL-guided BCP assembly defines the template, selective PMMA etching opens nanopores in PS, Au deposition provides seeds, CP-light photodeposition induces chiral Au growth, and lift-off leaves the guided nanoparticle array. Through subsequent circularly polarized light-induced Au photodeposition, the structures were endowed with chiral optical responses and could simultaneously encode multiple information dimensions, including orientation, Raman scattering, and chirality, thereby enabling multichannel optical anti-counterfeiting functionality [53].

Overall, DSA is expanding from conventional periodic pattern fabrication toward functional applications involving two-dimensional materials, three-dimensional nanostructures, and optoelectronic devices. Although most of these applications remain at the research stage, the role of DSA has extended beyond simple density multiplication toward the construction of controllable functional nanostructures.

Table 1 compares representative results using their reported dimensions, pattern-quality or electrical metrics, and demonstrated integration level.

## 4. Technical Challenges and Solutions

**Figure 8:**
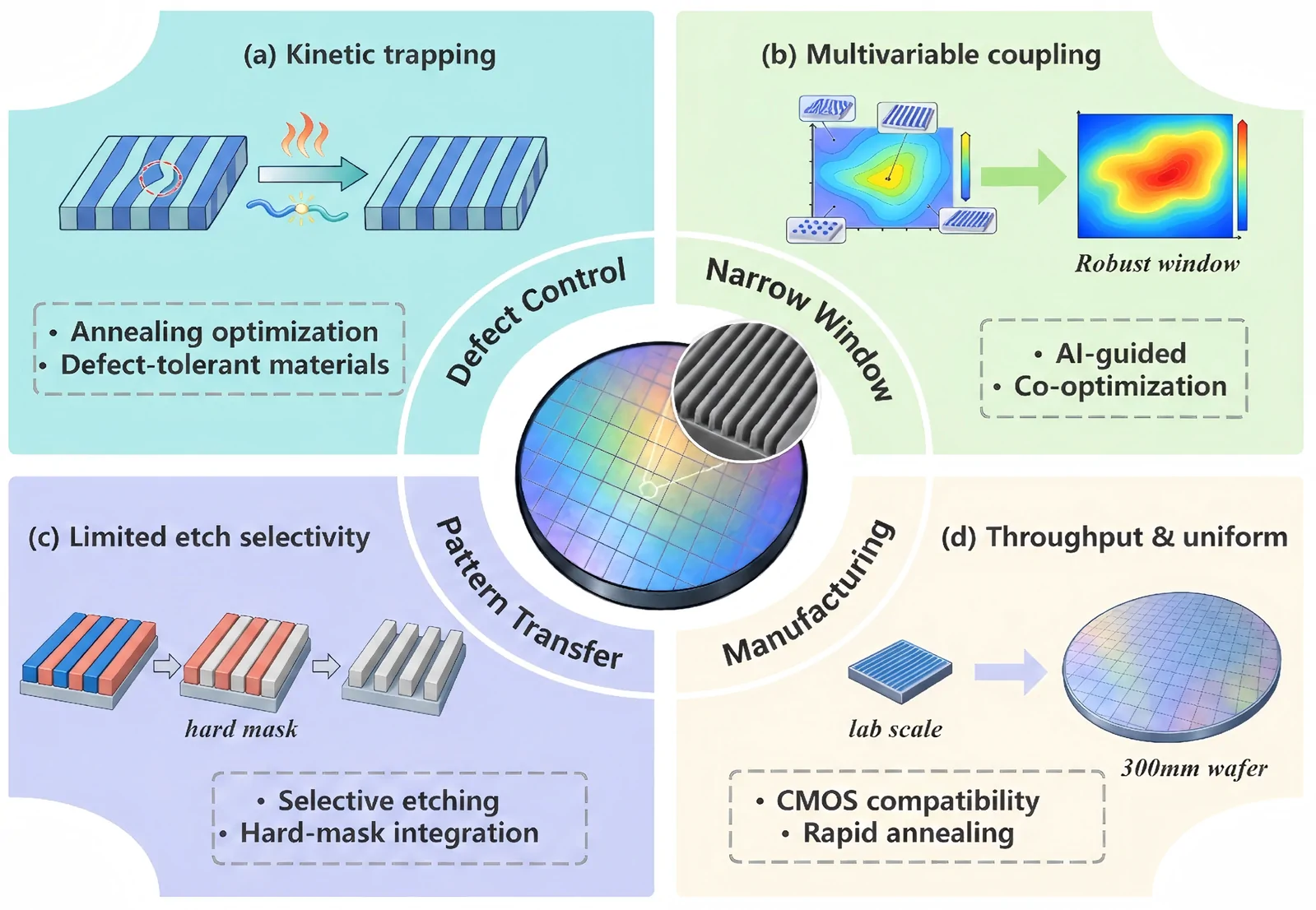
Overview of Technical Challenges and Solutions.

The transition of DSA from laboratory-scale demonstrations to wafer-scale manufacturing critically depends on translating its favorable self-assembly characteristics into stable, controllable, and reproducible process flows. Key challenges remain in defect control, process-window optimization, pattern transfer, and wafer-scale uniformity and throughput. To address these bottlenecks, ongoing efforts are focused on the systematic optimization of material systems, self-assembly kinetics, and manufacturing integration, as illustrated in Figure 8.

### 4.1. Defect Control

Defect density in DSA processes is a critical metric governing process feasibility. Defects such as dislocations, bridging, line breaks, and local domain loss are controlled not only by thermodynamic stability but also by kinetic factors, including polymer-chain diffusion, interfacial interactions, and confinement imposed by guiding templates. Consequently, even when the target structure is thermodynamically more stable, the system may remain trapped in metastable states because of kinetic barriers, resulting in residual defects.

For lamellar BCPs, dislocation removal relies on defect migration and annihilation. When dislocations are widely separated or the initial configuration is unfavorable, their migration may involve crossing multiple free-energy barriers and can be further constrained by chemical guiding patterns, leading to kinetic trapping [54,55]. Therefore, simply extending the annealing time does not necessarily guarantee a low-defect structure. Doise et al. demonstrated that, while maintaining sufficient microphase separation, appropriate tuning of χN together with an increased annealing temperature can accelerate defect annihilation in high-χ BCPs [54]. In addition, a correlation exists between the correlation length of unguided fingerprint patterns and the residual dislocation density after DSA, providing a useful auxiliary metric for evaluating assembly kinetics and screening annealing conditions.

A concrete failure mode is illustrated by Do et al. [8]: extending annealing from 300 to 1800 s at 280 °C worsened post-transfer LCDU and PPE by 28.9% and 21%, respectively, and produced merged holes. Thus, additional thermal budget can degrade patterns rather than improve ordering; the usable process window must be established for each material and stack.

In addition to kinetic regulation, the molecular architecture of BCPs can be engineered to improve tolerance to prepattern errors. Yun et al. compared the defect-rectification behavior of different BCP architectures, including linear AB diblock, A_1_B_1_A_2_B_2_ tetrablock, and comb-linear architectures, and found that molecular topology strongly influences process tolerance to edge roughness, dimensional mismatch, and pattern placement errors [56]. Certain complex architectures can broaden the guiding-parameter window while maintaining high pattern density, highlighting the potential of molecular design to improve the robustness of DSA processes.

Consistent with Section 2.1, thermodynamic stabilization of small domains by high-χ materials does not by itself ensure rapid ordering; the relevant segregation strength must remain compatible with defect relaxation on the available annealing timescale. Overall, defect control in DSA requires a balance among segregation strength, pattern resolution, and defect-annihilation kinetics, together with coordinated optimization of material design, guiding templates, and thermal-processing conditions. Future research should focus not only on reducing defect density but also on improving tolerance to process variations and prepattern errors, thereby enabling a more stable process window for wafer-scale manufacturing.

### 4.2. Process-Window and Multi-Parameter Co-Optimization

Another key challenge for DSA is that its process window is governed by the coupling of multiple factors, including material parameters, film conditions, annealing conditions, and guiding templates. The molecular weight, intrinsic period, and film thickness of the BCP, as well as the dimensions, pitch, and interfacial properties of the template, can all influence the resulting microphase-separated morphology, among which the commensurability between the template dimensions and the intrinsic BCP period is particularly critical. Deviations of these parameters from their suitable ranges can readily induce morphological transitions, positional shifts, and increased defectivity. Therefore, DSA optimization for advanced manufacturing should not be limited to identifying a single optimal process point, but should instead aim to establish a robust process window with a sufficiently broad tolerance range.

Early studies primarily relied on systematic experiments to determine the allowable ranges of BCP materials and template dimensions. Gharbi et al. investigated the PS-*b*-PMMA contact-hole shrink process on 300 mm wafers and found that, for a system with an intrinsic period of approximately 35 nm, guiding-hole dimensions of approximately 47–57 nm and pitches of approximately 100–130 nm enabled the stable formation of shrunk holes with a diameter of approximately 19.5 ± 1.2 nm. By adjusting the molecular weight, the final hole diameter could be further varied over a range of approximately 16–22 nm [57]. In addition, Latypov and Coskun introduced DSA-aware printing assist features to improve the tolerance of prepatterns to exposure variations [58], whereas Wu et al. tuned the self-assembly period by incorporating PMMA homopolymers with different molecular weights and blending ratios into PS-*b*-PMMA, thereby broadening the applicable template range for contact-hole shrinking from the perspective of material composition [21]. These studies demonstrate that the process window can be actively tuned through the co-design of guiding templates and BCP materials.

As the number of process variables and the complexity of their coupling increase, machine learning has begun to be employed for DSA morphology prediction and process-window optimization. Tu et al. used feedforward neural network and long short-term memory models to analyze film thickness, swelling degree, and annealing conditions in PS-*b*-PDMS systems, enabling prediction of different self-assembled morphologies with a test accuracy of approximately 80% [59]. Ginige et al. further combined quantitative defect analysis with support vector machines to identify low-defect regions within the parameter space defined by film thickness and swelling degree, thereby determining a relatively concentrated range of preferred processing conditions [60]. Compared with one-variable-at-a-time searches, data-driven approaches can more efficiently capture nonlinear relationships among process parameters and reduce the experimental search space.

For DSA to advance toward practical manufacturing, a key objective is to establish robust process windows that exhibit strong tolerance to variations in film thickness, template dimensions, annealing conditions, and material batches. At the same time, unified and well-defined standardized datasets are needed to provide a common basis for comparing results across different materials, equipment platforms, and process conditions. The integration of physics-based models, wafer-scale experimental data, and machine learning could further improve process-development efficiency and process-control capability in 300 mm wafer manufacturing.

### 4.3. Etch Selectivity and Pattern Transfer

Pattern transfer remains a major challenge in translating self-assembled DSA nanopatterns into functional device layers. For fully organic BCPs such as PS-*b*-PMMA, the intrinsic etch contrast between the constituent blocks is limited, while selective removal may also be accompanied by PS loss or swelling, thereby compromising critical-dimension control and pattern integrity. Recently developed highly selective wet-removal processes can enhance the PMMA/PS etch selectivity and further enable one-step PMMA removal without UV pretreatment, providing a promising route to reducing PS damage and simplifying the process flow [61].

Enhancing the intrinsic etch resistance of the template represents another important strategy. Si-containing BCPs can exploit their inorganic components to improve etch resistance and pattern height [26], whereas SIS can selectively form inorganic hard masks such as ${\mathrm{Al}}_{2}O_{3}$ within specific BCP domains, thereby enhancing subsequent pattern-transfer capability [9]. However, pattern transfer is governed not only by hard-mask robustness but can also alter the spectral characteristics of LER, LWR, and pattern-placement roughness. A comparison of SIS and dry lift-off by Loo et al. showed that different transfer strategies exhibit distinct roughness evolution across different spatial scales. Therefore, pattern-transfer performance cannot be evaluated solely on the basis of overall etch selectivity [62].

Accordingly, future DSA pattern-transfer processes must balance etch selectivity, critical-dimension preservation, roughness control, and process complexity. Strategies including low-damage, highly selective block removal, inorganic-containing BCPs with intrinsically high etch resistance, and SIS-enhanced hard masks should be further co-optimized with highly anisotropic etching and multilayer hard-mask stacks to improve the stability and reproducibility of transferring sub-10 nm patterns into functional device layers.

### 4.4. Challenges for High-Volume Manufacturing

In recent years, high-χ BCPs and rapid annealing strategies have continued to advance. However, achieving shorter assembly times while maintaining low defect density, well-controlled orientation, and high pattern fidelity remains a key challenge for improving manufacturing efficiency [63].

Achieving low defectivity, high uniformity, and good reproducibility at the 300 mm wafer scale is equally critical. Variations in BCP molecular-weight distribution, film thickness, interfacial chemistry, template dimensions, and annealing conditions can all induce local deviations in periodicity and structural defects. Therefore, wafer-scale DSA requires the integration of material and template optimization, high-precision process control, and highly sensitive defect metrology to improve process stability [5].

The industrial value of DSA also depends on its overall cost and process complexity. High-purity BCPs, dedicated guiding layers, annealing steps, and defect inspection can all increase the process burden. Its practical competitiveness must therefore be evaluated comprehensively across the entire manufacturing chain, including materials, equipment, metrology, design rules, and yield [64]. In addition, DSA is more likel y to serve as a complementary technology to EUV lithography in industrial applications. Its adoption in high-volume manufacturing will ultimately depend on achieving an overall balance among resolution, defectivity, throughput, cost, and compatibility with CMOS/EUV process flows.

Scale-up should therefore be assessed after pattern transfer and device integration, using wafer maps and cross-lot distributions of defects and critical dimensions together with overlay, throughput, and electrical yield. Longer annealing or additional guiding and transfer steps need to be justified by their net benefit within the complete process flow.

## 5. Summary and Outlook

Directed self-assembly exploits the controlled microphase separation of BCPs to generate sub-lithographic nanoscale patterns and represents a promising complementary patterning technology for advanced semiconductor manufacturing. This review summarizes the mechanisms of BCP microphase separation, the major guiding strategies including graphoepitaxy and chemoepitaxy, and hybrid patterning approaches integrating DSA with deep ultraviolet (DUV)/EUV lithography. In recent years, the role of DSA has expanded from conventional density multiplication to pattern rectification, the fabrication of high-density regular structures, and the modulation of functional nanostructures, with demonstrations in FinFETs, DRAM, ReRAM, PCM, two-dimensional semiconductors, three-dimensional interconnected structures, and CMOS optoelectronic devices.

Although DSA patterning and integration have been demonstrated on 300 mm wafers, these demonstrations do not by themselves establish high-volume manufacturing qualification. Adoption remains constrained by defect-annihilation kinetics, strongly coupled process windows, pattern-transfer limitations, and challenges in wafer-scale uniformity, throughput, defect metrology, yield, and reproducibility.Further progress therefore requires coordinated optimization of BCP materials, guiding templates, annealing and self-assembly kinetics, pattern transfer, and metrology, with greater emphasis on process tolerance to material and process variations rather than optimization at a single process point.

EUV–DSA hybrid patterning is likely to remain one of the most practically relevant development directions in the near term. Under appropriate conditions, the ordering process associated with BCP microphase separation can mitigate roughness, local dimensional variations, and certain defects arising from EUV stochastic effects [65,66]. Meanwhile, advances in high-χ BCPs, Si-containing BCPs, and materials with high etch contrast are expected to further improve sub-10 nm patterning and subsequent pattern-transfer capability [13,67]. In parallel, inverse design, computational simulation, and automated parameter optimization can be used to resolve the multivariable coupling among film thickness, annealing conditions, template dimensions, and interfacial properties, thereby facilitating the identification of robust process windows with low defectivity and greater tolerance [68,69]. Further integration with DSA-aware design tools and high-throughput defect metrology may enable more effective co-optimization of materials, processes, and layout design.

For emerging three-dimensional logic architectures such as gate-all-around field-effect transistors (GAAFETs) and complementary field-effect transistors (CFETs), DSA is more likely to be initially applied to highly regular patterning layers, including high-density contact holes, periodic line/hole arrays, local interconnects, and other repetitive structures, rather than directly undertaking fabrication of complete device architectures. Direct device-level demonstrations remain limited, and practical implementation will depend on the compatibility of DSA with EUV lithography, highly selective pattern transfer, multilayer overlay, and three-dimensional integration processes. Overall, a substantial technological gap remains before widespread high-volume manufacturing can be achieved. Nevertheless, continued advances in high-χ BCPs, advanced metrology, computational design, and three-dimensional integration may establish DSA as an important complement to advanced lithography and provide additional patterning pathways for semiconductor devices with smaller dimensions and increasingly complex architectures.

## Figures and Tables

**Table 1:** Quantitative benchmarks and demonstrated integration levels of representative DSA studies.

Route/Target	Geometry/Scale	Reported Quantitative Result
High-χ chemoepitaxy; line patterns [6]	<10 nm half-pitch; 300 mm wafers	Perpendicular lamellae with reduced parallel regions and dislocations
Chemoepitaxial FinFET fins [7]	BCP period 25–30 nm; 300 mm platform	Median $V_{T,\mathrm{lin}}$: 0.176 vs. 0.166 V; $SS_{\mathrm{lin}}$: 62.1 vs. 62.9 mV/dec (DSA vs. SAQP, $L_{g}\;\;\approx \;\;500$ nm)
EUV–DSA contact rectification [8]	Hexagonal holes; 30 nm pitch	LCDU improved 62.5%, PPE 22.8% before transfer; separate 250 °C/1800 s split: 1 defect in 54,000 $\mu m^{2}$
DSA–SIS silicon JL-FinFETs [9]	Top width 6.6 nm; pitch 28 nm; height 53.5 nm	SS=69.59 mV/dec; $I_{\mathrm{on}}/I_{\mathrm{off}}>10^{7}$ ($V_{D}=0.1$ V; $L_{\mathrm{ch}}=3$ μm)
Trimmed honeycomb guides; DRAM-relevant arrays [10]	BCP period 28 nm	Total areal multiplication 48×=2×24: template trimming, then BCP assembly
CMOS image-sensor optical interface [11]	DSA mask hole diameter ∼15 nm; pitch ∼40 nm	Quantum-efficiency improvement ∼9% (blue), ∼4% (green); comparable dark current after etch optimization

## Data Availability

No new data were created or analyzed in this study. Data sharing is not applicable to this article.

## Abbreviations

The following abbreviations are used in this manuscript:

BCC	Body-centered cubic
BCP	Block copolymer
BPM	Bit-patterned media
CD	Critical dimension
CDU	Critical dimension uniformity
CMOS	Complementary metal–oxide–semiconductor
DRAM	Dynamic random-access memory
DSA	Directed self-assembly
EBL	Electron beam lithography
EUV	Extreme ultraviolet
FET	Field-effect transistor
FinFET	Fin field-effect transistor
GNR	Graphene nanoribbon
GYR	Gyroid
HEX	Hexagonally packed cylindrical phase
HSQ	Hydrogen silsesquioxane
ICP	Inductively coupled plasma
LAM	Lamellar phase
LCDU	Local critical dimension uniformity
LER	Line-edge roughness
L/S	Line/space
LWR	Line-width roughness
NIL	Nanoimprint lithography
PCM	Phase-change memory
PPE	Pattern placement error
pHRF	Pseudo-high-refractive-index film
PS-*b*-PDMS	Polystyrene-block-poly(dimethylsiloxane)
PS-*b*-PMMA	Polystyrene-block-poly(methyl methacrylate)
ReRAM	Resistive random-access memory
RIE	Reactive ion etching
SAQP	Self-aligned quadruple patterning
SCFT	Self-consistent field theory
SiNW	Silicon nanowire
SIS	Sequential infiltration synthesis
SOI	Silicon-on-insulator
UV	Ultraviolet
